# An assessment of the maxilla after rapid maxillary expansion using cone beam computed tomography in growing children

**DOI:** 10.1590/2176-9451.19.1.026-035.oar

**Published:** 2014

**Authors:** Jessica L. Woller, Ki Beom Kim, Rolf G. Behrents, Peter H. Buschang

**Affiliations:** 1 Former resident, Department of Orthodontics, Center for Advanced Dental Education, Saint Louis University.; 2 Assistant professor, Department of Orthodontics, Saint Louis University.; 3 Professor, Head of the Department of Orthodontics, Saint Louis University.; 4 Adjunct professor, Department of Orthodontics, Saint Louis University and Baylor University.

**Keywords:** Palatal expansion technique, Orthodontics, Cone beam computed tomography, Cranial sutures

## Abstract

**Introduction:**

With the advent of cone beam computed tomography (CBCT), it is now possible to quantitatively evaluate the effects of rapid maxillary expansion (RME) on the entire maxillary complex in growing patients.

**Objective:**

The purpose of this study is to use three-dimensional images to evaluate the displacement that occurs at the circummaxillary sutures (frontonasal, zygomaticomaxillary, intermaxillary, midpalatal, and transpalatal sutures) following rapid maxillary expansion in growing children.

**Methods:**

The CBCT scans of 25 consecutively treated RME patients (10 male, 15 female) with mean age of 12.3 ± 2.6 years, were examined before expansion and immediately following the last activation of the expansion appliance.

**Results:**

Statistically significant (P < 0.05) amounts of separation were found for the displacement of the bones of the frontonasal suture, the intermaxillary suture, the zygomaticomaxillary sutures, and the midpalatal suture. The change in angulation of the maxillary first molars due to RME was also statistically significant. There was no statistically significant displacement of the transpalatal suture.

**Conclusions:**

Rapid maxillary expansion results in significant displacement of the bones of circummaxillary sutures in growing children.

## Abstract (pt)

**Introdução:**

com o advento da tomografia computadorizada de feixe cônico (TCFC), hoje é possível avaliar quantitativamente os efeitos da expansão rápida da maxila (ERM) em todo o complexo maxilar em pacientes em crescimento.

**Objetivo:**

este estudo usou imagens tridimensionais para avaliar o deslocamento das suturas circum-maxilares (frontonasal, intermaxilar, palatina mediana e palatina transversa) após a expansão rápida da maxila em crianças em crescimento.

**Métodos:**

imagens de TCFC de 25 pacientes tratados consecutivamente com ERM (10 meninos, 15 meninas) com idade média de 12,3 ± 2,6 anos foram obtidas e examinadas antes da expansão e imediatamente após a última ativação do aparelho expansor.

**Resultados:**

houve deslocamento estatisticamente significativo (p < 0,05) dos ossos da sutura frontonasal, intermaxilar, zigomaticomaxilar e palatina mediana. A alteração na angulação dos primeiros molares superiores devido à ERM também foi estatisticamente significativa. Não houve deslocamento estatisticamente significativo da sutura palatina transversa.

**Conclusões:**

a expansão rápida da maxila resultou em deslocamentos significativos dos ossos das suturas circum-maxilares em crianças em crescimento.

## INTRODUCTION

Rapid maxillary expansion (RME) is the most common orthopedic procedure used to correct a maxilla with transverse discrepancy. Heavy orthopedic forces are used to separate the two halves of the maxilla at the midpalatal suture.^1^ Indications for RME include the need to correct posterior crossbite and to increase arch perimeter in patients with a tooth-size arch-length deficiency to address crowding.^2,3^

Along with the opening of the midpalatal suture,^4,5^ RME has an effect on the entire maxillary complex.^6-9^ According to Starnbach et al,^10^ palatal expansion does not only separate the midpalatal suture, but the circumzygomatic and circummaxillary sutural systems as well. Specifically, the nasal, the zygomaticomaxillary, and the zygomaticotemporal are some of the sutures affected by RME. Studies employing dry skulls,^7^ rhesus monkeys^11-14^ and finite element models (FEM)^15,16,17^ have proved these sutures to be affected, but the descriptions tend to be qualitative in nature, except for the stress levels calculated by the FEM studies.

While there are significant contributions from all of these past studies, there are weaknesses associated with each type of study mentioned above. Studies on dry skulls lack the soft tissues that can hamper the effects of RME, particularly the connective tissue that forms the suture between bones. Rhesus monkeys have a significantly different maxillary anatomy in comparison to humans. The biggest problem FEM faces is the fact that the computer program used for the study is only as good as the model upon which it is based. FEM studies that examined RME were based upon one dry human skull each. Additionally, FEM studies do not demonstrate longitudinal effects, only a particular instant in time.

Many studies^5,8,18-29^ have attempted to quantify the changes that occur in the maxillary complex as a result of RME. Several studies have extensively reviewed the changes occurring at the midpalatal suture, the dentoalveolar structures, and the nasal cavities.^9,25,28,30-33^ However, the limitations of clinical examinations and two-dimensional radiography inhibit the analysis of what is occurring at the sutural levels in orthodontic patients. The introduction of cone beam computed tomography (CBCT) in Orthodontics^34,35^ now permits the examination of the craniofacial complex in living, growing subjects.

The purpose of this study is to use three-dimensional images to evaluate the changes that occur at the circummaxillary sutures, including the frontonasal, zygomaticomaxillary, intermaxillary, midpalatal, and transpalatal ones, following rapid maxillary expansion in growing children. In addition, the relationship between the midpalatal suture and the other sutures are also analyzed.

## MATERIAL AND METHODS

### Patient selection

This investigation is a retrospective study approved by the Saint Louis University Institutional Review Board (#15727). The records of 25 consecutively treated patients (10 male, 15 female) were chosen from the records of private practice based on the following selection criteria:

### Inclusion criteria

1. Diagnosis included a finding of skeletal transverse discrepancy, while treatment plan included the use of a rapid maxillary expansion appliance.

2. Complete set of CBCT images including one prior to appliance delivery and one taken immediately after the active expansion phase of treatment.

### Exclusion criteria

1. Patients with craniofacial anomalies, including cleft lip and palate.

2. Patients with orthodontic appliances present prior to the start of treatment with rapid maxillary expansion.

Patients' mean age at the time of the first imaging appointment in this study was 12.3 ± 2.6 (8.3 to 17.8 years). The second CBCT image was taken on an average of 22.8 ± 5 days after the first image (14 to 37 days).

Each patient was treated with a tooth-borne rapid maxillary expander (Hyrax). The expansion appliance consisted of a 7-millimeter Dentaurum expansion screw (Dentaurum, Ispringen, Germany) with 0.051-inch diameter stainless steel arms welded to orthodontic bands on the maxillary first molars, and a 0.051-inch diameter supporting wire placed palatal to the dentition and bands so as to increase the rigidity of the appliance and extend the force of the expander to the canines as well as first and second premolars, if they were present (Fig 3).

**Figure 1:**
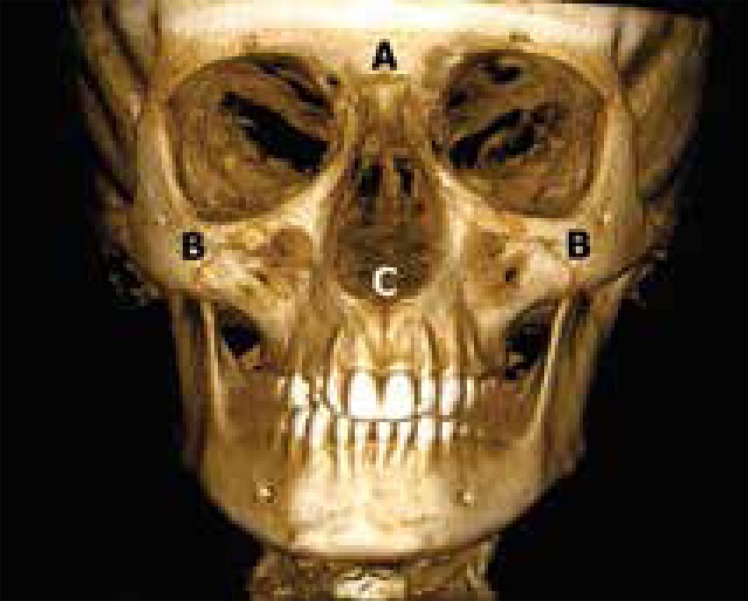
Frontal skull showing evaluated areas.

**Figure 2:**
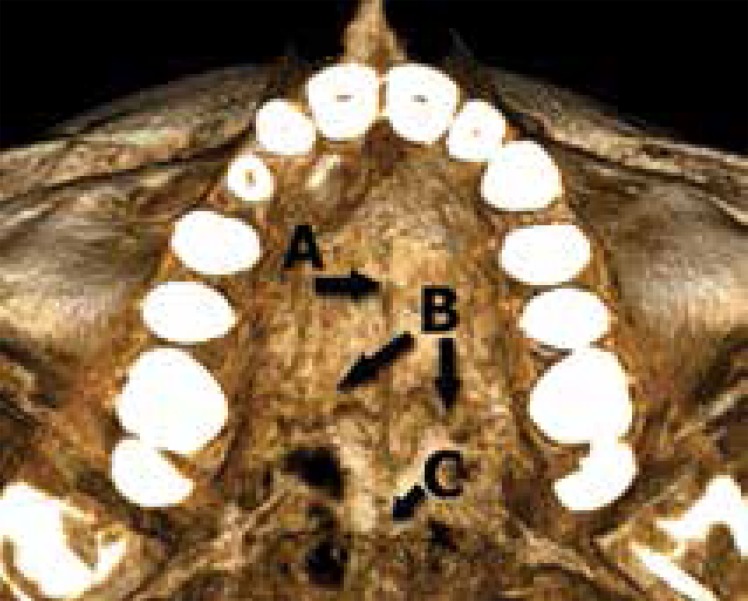
Axial view of a skull showing evaluated areas. A) Midpalatal suture; B) Transpalatal suture; C) PNS.

**Figure 3:**
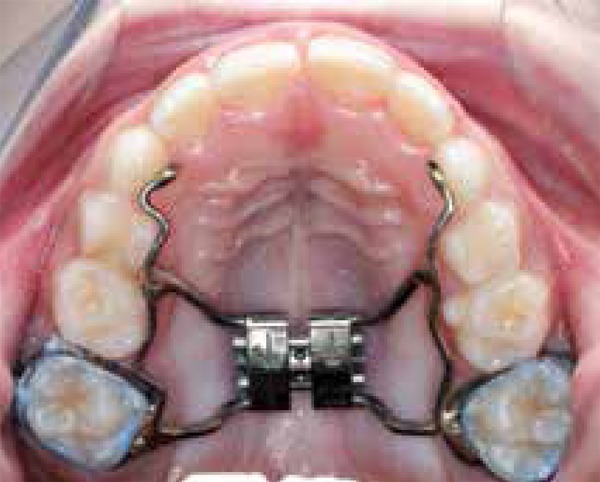
Model of palatal expander.

The expander was activated two-quarter turns of the expansion screw (0.2 mm each turn) at the time of delivery, followed by a one-quarter turn twice a day. Activation of the screw continued until the transverse discrepancy was overcorrected to the point in which the palatal cusps of the maxillary molars were in edge-to-edge contact with the buccal cusps of the opposing mandibular teeth.

### Imaging

Cone beam computed tomography scans were taken using the Classic i-CAT^®^ (Imaging Sciences International, Inc., Hatfield, USA) cone beam CT scanner. All scans were taken by the same technician using either the 16 x 13 or the 16 x 22 centimeter field of view with a voxel size of 0.4 millimeters. Patients were positioned in a vertical seat with their head stabilized in the headrest to prevent any unwanted movement during the 20-second scan, teeth together in centric occlusion, and the Frankfort Horizontal plane parallel to the floor, as determined by the external auditory meatus and soft-tissue orbitale.

Each patient was scanned at two different time points: T_0_ and T_1_. The first image (T_0_) was obtained prior to the delivery of the expander and represented the subject's baseline condition prior to expansion. The second time point (T_1_) was taken at the appointment immediately following the last activation of the expansion appliance.

The analysis measured changes of the alveolar bone and maxillary sutures following rapid maxillary expansion with consistent landmark identification using the Dolphin 3-D software (Dolphin Imaging & Management Solutions, Chatsworth, USA).

**Figure 4:**
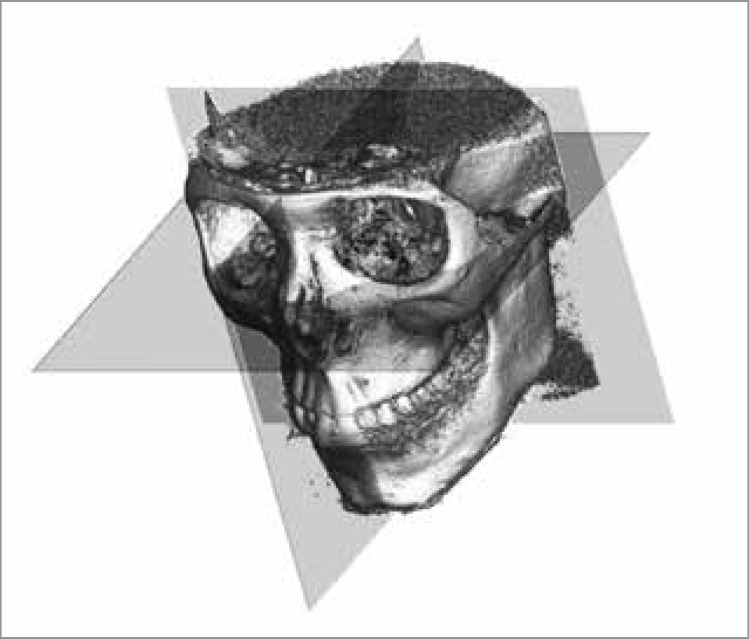
CBCT image after orientation.

Each scan had a number randomly assigned and loaded into the three-dimensional software so that each scan was analyzed without the operator identifying the patient. First, each scan was oriented by locating the midpoint between both foramina spinosum (ELSA), and assigning to it x = 0, y = 0, and z = 0 coordinates (Fig 4). The following points were then located: 1) the superior-lateral border of the external auditory meatus (SLEAM) on both right and left sides, and 2) the mid-dorsum of the foramen magnum (MDFM). An axial-horizontal plane (x-y plane) was determined by using the right and left SLEAM points and ELSA. A sagittal-vertical plane (z-y plane) was determined perpendicular to the x-y plane and passing through points ELSA and MDFM. These points have shown a high intra-reliability when located with 3D images, which makes the x-y and z-y planes formed by these points an adequate way to standardize the orientation of 3D images.

## Landmark location

Two-dimensional axial images were created perpendicular to the coronal plane and used to measure the amount of midpalatal and transpalatal suture separation on the external surface of each suture.

The midpalatal suture was measured adjacent to four locations: the first molar, the contact area between the first and second premolars, the canine, and the most anterior point of the maxillary dental arch.

**Figure 5:**
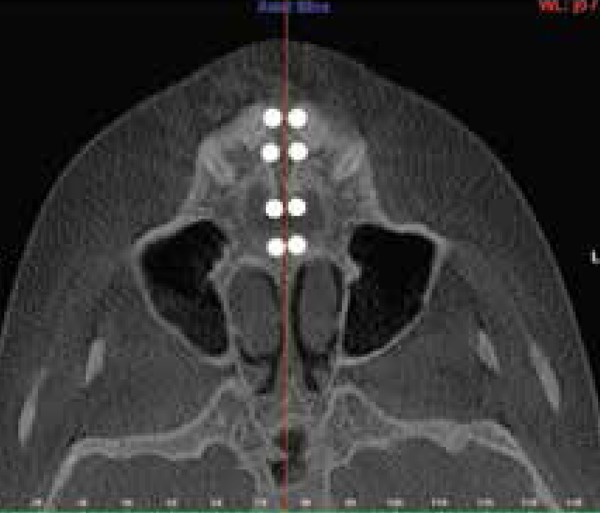
Axial slice through the palate showing the midpalatal suture.

The central groove of each first molar was identified by locating the crown of the molar on an axial image and marking the central groove. An axial section through the hard palate was then created, and the mesial edge of the midpalatal suture was marked on both right and left sides. The same procedure was followed for the other areas of the midpalatal suture, as described above (Fig 5). To verify if the external surface of the suture was being marked, coronal slices were viewed for the places where the suture edges were marked and corrected if necessary.

**Figure 6:**
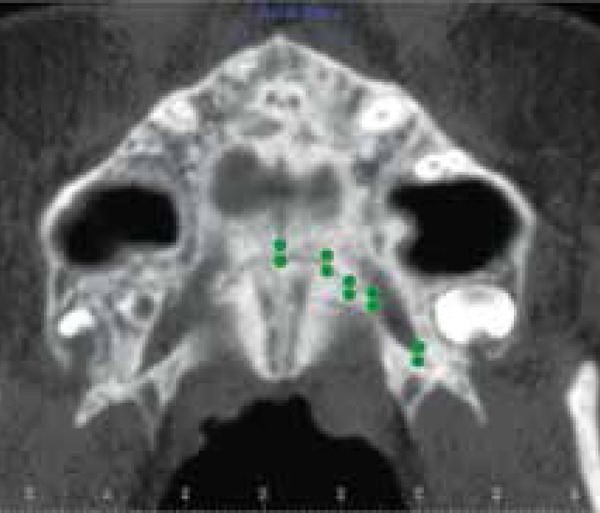
Axial slice through the palate showing the transpalatal suture.

The transpalatal suture was measured at five areas along the suture. The first area identified was in the midline, established by the junction of the midpalatal and transpalatal sutures. The proximal and distal edges of the transpalatal suture were marked at the midline, as well as at 6, 12, and 18 millimeters to the left (Fig 6). The millimetric grid on the axial image was used to determine the landmarks to the left of the midline. To verify if the external surface of the suture was being marked, coronal slices were viewed for the places where the suture edges were marked and corrected if necessary.

**Figure 7:**
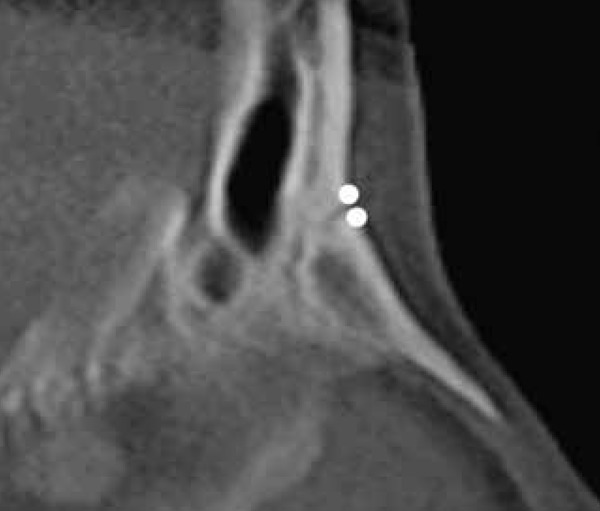
Sagittal slice through the midline, with the frontonasal suture marked.

The displacement of the frontonasal suture was determined by locating and marking the superior and inferior edges of the frontonasal suture on the external surface in the midline on a sagittal section (Fig 7).

**Figure 8:**
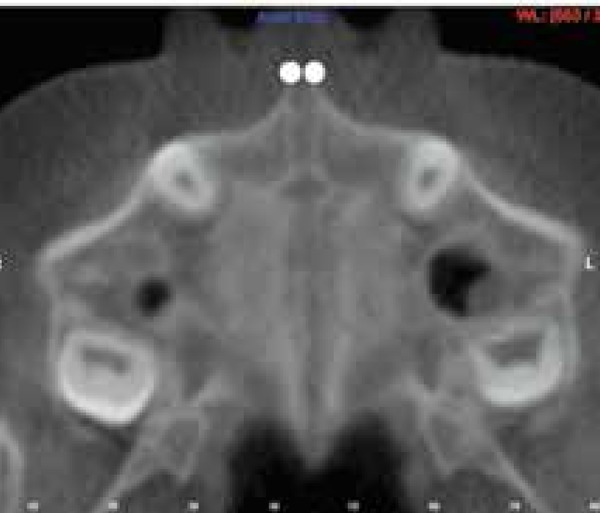
Axial slice through the hard palate, with the intermaxillary suture marked at ANS on the right and left sides.

**Figure 9:**
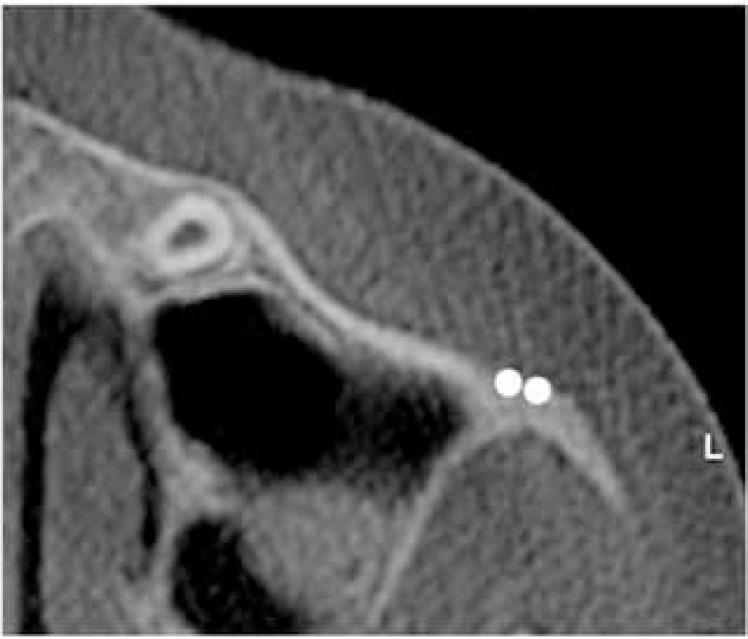
Axial slice showing the left inferior border zygomaticomaxillary suture.

**Figure 10:**
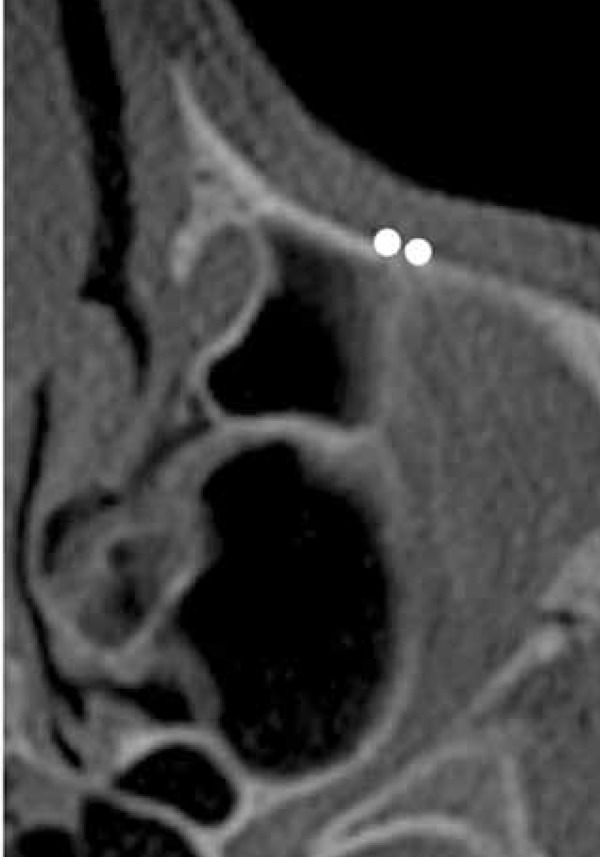
Axial slice showing the left superior border of zygomaticomaxillary suture.

Axial sections were used in the appropriate locations to locate and measure the mesial edges of the intermaxillary suture at ANS (Fig 8) as well as the proximal and distal edges of the zygomaticomaxillary sutures which were located and marked on both inferior (Fig 9) and superior (Fig 10) borders of the zygomaticomaxillary suture on both right and left sides.

**Figure 11:**
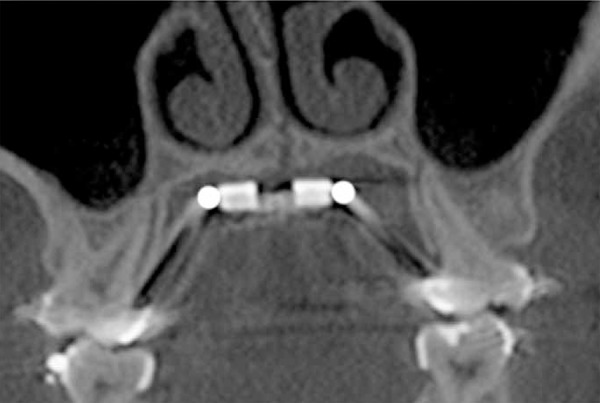
Post-expansion coronal slice at the central groove of the maxillary first molar, with the expansion screw in the center.

To determine the amount of appliance expansion, the outer edges of an unactivated 7-mm Dentaurum expansion screw (Dentaurum, Ispringen, Germany) were measured with digital calipers. In the post-expansion scans, the outer edges of the expansion screw were marked in the coronal slice at the maxillary first molars (Fig 11).

**Figure 12:**
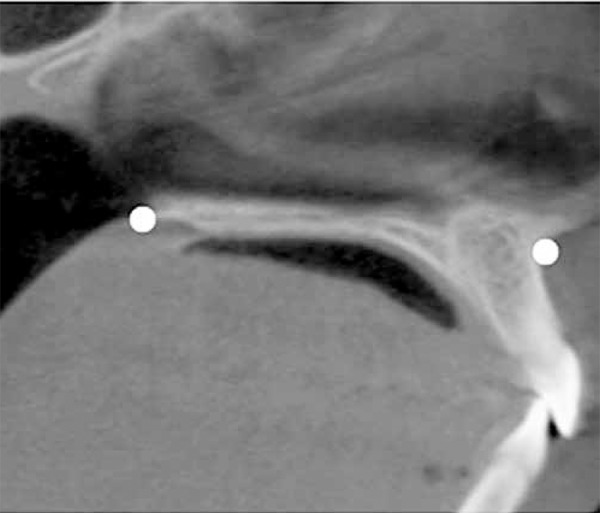
A point (right) and PNS (left) on a sagittal slice through the midline.

The A point and the posterior nasal spine (PNS) were identified on a sagittal section (Fig 12).

**Figure 13:**
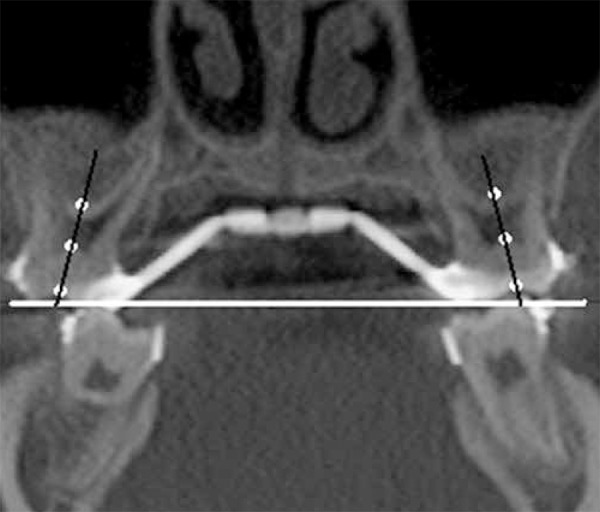
Angulation of the maxillary first molars measured at the occlusal plane.

To establish the amount of maxillary first molar angulation, the long axis of each first molar was determined by identifying the center of the pulp chamber on axial slices at several levels. Then, the angle of the each molar was calculated to the occlusal plane (Fig 13).

For each landmark described above, the x, y, and z coordinates were recorded at both T_0_ and T_1_ for each patient. The value in millimeters for each coordinate was entered into an Excel software spreadsheet (Microsoft, Redmond, Washington, USA). To calculate the width of each suture, the distance between the two edges was calculated for each coordinate. To calculate the amount of expansion, the difference between the right and left sides of the expansion jackscrew was computed for each coordinate. Analytical geometry was used to convert each set of coordinates into a single value. The resulting value was the width of a suture at a particular location or the amount of appliance expansion. These calculations were done for all of the x, y, and z coordinates for both time intervals.

### Statistics

All statistics were calculated using the SPSS 14.0 Statistical Software (SPSS Inc., Chicago, Illinois, USA) on a personal computer. Descriptive statistics were calculated for all suture width measurements and for appliance expansion as well as maxillary first molar angulation and change in position of A point and PNS.

To calculate intra-examiner reliability, 25% of the CBCT scans were re-measured to test the reliability of landmark identification with an intraclass correlation coefficient. Reliability of scan orientation was also calculated using the intraclass correlation coefficient by re-orienting 25% of the CBCT scans and using the cranial base landmarks basion and anterior clinoid process of sella turcica for comparison.

Non-parametric statistics were used due to the small sample size.^37^ Changes in measurements between pre and post expansion for each of the sutures were analyzed using single-tailed Wilcoxon signed rank tests with a level of significance set at P < 0.05. Kendall's Tau-b correlations with a significance level set at P < 0.05 were calculated to assess the relationships between the various sutures and the amount of expansion.

## RESULTS

The intraclass correlation coefficient proved the measurements and orientation to be very reliable. Cronbach's Alpha measurement of 0.947 showed that there was no significant difference between the original measurements made and the repeated measurements performed on a sample that was randomly selected and represented in 25% of the sample. Likewise, Cronbach's Alpha measurement of 0.967 showed that there was no significant difference between the original orientation made and the repeated orientation performed on a sample that was randomly selected and represented in 25% of the sample. 

Descriptive statistics for the changes in suture width measurements between T_0_ and T_1_ as well as the amount of appliance expansion are listed in Table 1.

Statistically significant differences were found for the sutural displacement of the frontonasal suture (z= -3.714, P < 0.001), the right superior zygomaticomaxillary suture (z= -3.951, P < 0.001), the right inferior zygomaticomaxillary suture (z= -2.677, P < 0.007), the left superior zygomaticomaxillary suture (z= -3.415, P < 0.001), the left inferior zygomaticomaxillary suture (z= -3.633, P < 0.001), the intermaxillary suture, as measured at ANS (z= -4.200, P < 0.001), and all landmarks along the midpalatal suture (P < 0.001). There was no statistically significant displacement regarding the transpalatal suture at any of the measured landmarks. A point had a statistically significant movement after RME, however, PNS did not, even though it had 1.03 millimeters of movement downward.

**Table 1:** Descriptive statistics of sutural displacement (T1-T0).

Measurement (mm)	n	Minimum Value	Maximum Value	Mean ± SD
Appliance expansion	25	1.8	6.8	4.2 ± 1.1*
Frontonasal suture displacement	25	0.1	1.8	0.6 ± 0.4*
Intermaxillary suture displacement	25	0.0	3.1	1.9 ± 0.8*
Right superior zygomaticomaxillary suture displacement	25	0.3	3.6	1.2 ± 0.9*
Left superior zygomaticomaxillary suture displacement	25	0.0	1.7	0.6 ± 0.4*
Right inferior zygomaticomaxillary suture displacement	25	0.4	4.2	1.1 ± 0.9*
Left inferior zygomaticomaxillary suture displacement	25	0.4	3.7	1.2 ± 0.8*
Midpalatal suture displacement at maxillary first molar	25	0.1	2.0	0.9 ± 0.5*
Midpalatal suture displacement at contact between maxillary first and second premolar	25	0.1	2.4	0.8 ± 0.6*
Midpalatal suture displacement at maxillary canine	25	0.2	2.8	1.1 ± 0.7*
Midpalatal suture displacement at the most anterior point of the maxillary dental arch	25	0.5	3.0	1.5 ± 0.6*
Transpalatal suture displacement at the palatal midline	25	0.3	3.3	1.2 ± 0.8
Transpalatal suture displacement 6 mm to left to palatal midline	25	0.1	1.5	0.7 ± 0.4
Transpalatal suture displacement 12 mm to left to palatal midline	25	0.1	2.3	0.7 ± 0.5
Transpalatal suture displacement 18 mm to left to palatal midline	25	0.0	2.8	0.6 ± 0.6

* Indicates displacement is statistically significant (p< 0.05)

**Figure 14:**
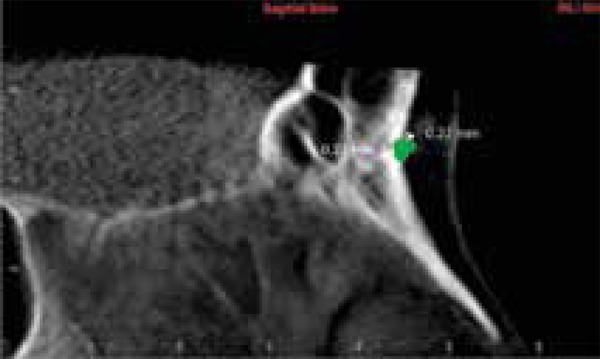
Frontonasal suture movements in Y and Z planes (sagittal slice).

**Figure 15:**
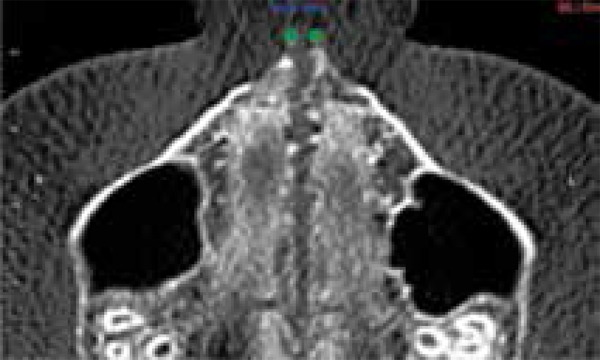
Intermaxillary suture movement at ANS in X plane (axial slice).

**Figure 16:**
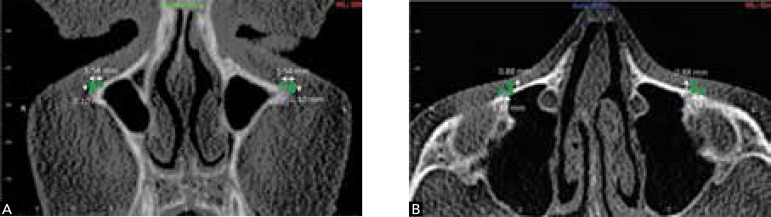
Superior zygomaticomaxillary suture movements in A) X and Y planes (coronal slice) and B) X and Z planes (axial slice).

**Figure 17:**
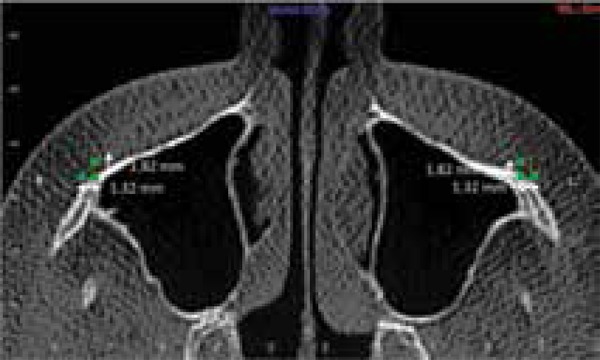
Inferior zygomaticomaxillary suture movements in X and Z planes (axial slice).

**Figure 18:**
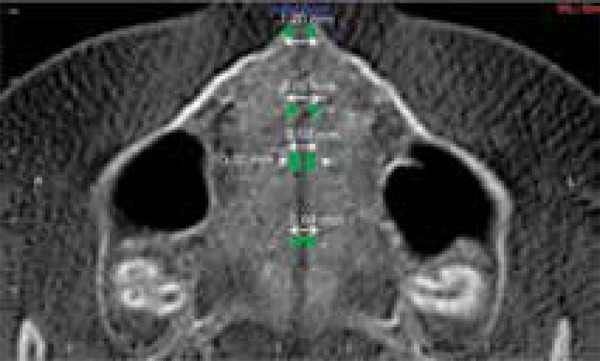
Midpalatal suture movements in X and Z planes (axial slice).

**Figure 19:**
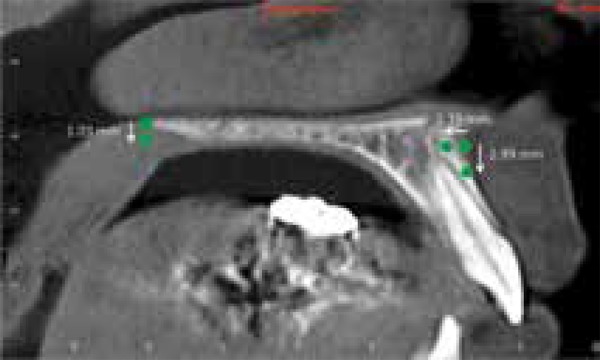
Movements in Y and Z planes (sagittal slice) of A point (right) and PNS (left).

When the individual x, y, and z coordinates were evaluated, the movement of the edges of the sutures was found to be significant in some directions. The results are listed in Table 2. Figures 14 to 18 demonstrate the significant movements for each assessed suture. Figure 19 demonstrates the movement of A point and PNS.

**Table 2:** Significant movement of individual coordinates.

		X	Y	Z
Frontonasal suture	z value		-3.115	-2.642
	sig (one-tailed)	NS	0.001	0.004
	movement (mm)		-0.33	0.22
Intermaxillary suture (ANS)	z value	-4.203		
	sig (one-tailed)	0.001	NS	NS
	movement (mm)	1.82		
Superior border of zygomaticomaxillary suture	z value	-5.505	-2.894	-6.037
	sig (one-tailed)	0.001	0.002	0.001
	movement (mm)	1.54	-0.1	0.88
Inferior border of zygomaticomaxillary suture	z value	-5.475		-5.761
	sig (one-tailed)	0.001	NS	0.001
	movement (mm)	1.32		1.82
Midpalatal suture at maxillary first molar	z value	-3.46	-2.236	
	sig (one-tailed)	0.001	0.01	NS
	movement (mm)	0.64	-0.02	
Midpalatal suture at contact of maxillary first and second premolar	z value	-3.353		-2.432
	sig (one-tailed)	0.001	NS	0.008
	movement (mm)	0.6		-0.12
Midpalatal suture at maxillary canine	z value	-3.742	-1.732	
	sig (one-tailed)	0.001	0.04	NS
	movement (mm)	0.86	-0.01	
Midpalatal suture at the most anterior point of the dental arch	z value	-4.186		
	sig (one-tailed)	0.001	NS	NS
	movement (mm)	1.2		
A point	z value			-1.672
	sig (one-tailed)	NS	NS	0.05
	movement (mm)		-2.99	-1.39

Kendall's correlations showed weak relationships between the midpalatal suture at the first molar and the frontonasal suture (t = 0.261, P < 0.036) as well as the superior border of the zygomaticomaxillary suture and the midpalatal suture at: the most anterior aspect of the dental arch (t = 0.418, P < 0.002), the contact between first and second premolars (t = 0.322, P < 0.012), and at the first molar (t = 0.364, P < 0.006). There was also a weak relationship between the inferior border of the zygomaticomaxillary suture and the midpalatal suture at the most anterior aspect of the dental arch (t = 0.261, P < 0.034).

The difference in angulation of the maxillary first molars to the occlusal plane was statistically significant (right: z = -3.135, P < 0.001, left: z = -3.586, P < 0.001) before and after RME. Prior to RME, the right first molar had an average angulation of 87.3° while the left first molar had an average angulation of 84.4°. After RME, the right first molars crown tipped buccally with an average resulting angle of 82.3*° *and the left first molars crown also tipped buccally with an average resulting angle of 78.8°. Using geometry to calculate the intramolar angle, the pre-RME angle between right and left maxillary first molars was 12.3° and after RME it increased to 22.7°.

## DISCUSSION

To date, there are a number of methods for studying the effects of rapid maxillary expansion. However, the literature does not reach a consensus regarding which approach is superior. In addition, the use of non-distorted three-dimensional imaging is a relatively new technique being in Orthodontics used to quantify the skeletal and dental effects of rapid maxillary expansion.

Although there are many studies^5,8,18-28^ regarding rapid maxillary expansion, very few have used cone beam computed tomography technology.^25^

The sample size of 25 was substantially larger than the one used in many other three-dimensional studies. This gave more power to the statistical analyses to reveal significant differences when they existed. This sample was also unique in the fact that the CBCT images were taken relatively close together, with the second image occurring shortly after the active expansion of the appliance was finished. This eliminates most of the remodeling and growth, as well as relapse that appears in other studies.

The midpalatal suture exhibited significant displacement due to RME, with the amount of displacement being greater in the anterior than in the posterior. As this result is consistent with previous studies,^5,8,18-28^ the displacement of other sutures was of more interest for this study. Many studies have concluded that the palatal shelves tip down as well as move apart transversely during RME. In this study, only the transverse movement was shown to be significant along the midpalatal sutures edges. There was significant downward movement at the contact of the first and second premolars, but the amount was only a fraction of a millimeter.

In studies carried out with rhesus monkeys, Starnbach^10^ noted that during RME, there was increased cellular activity at the zygomaticomaxillary suture, indicating bone formation at that suture. In contrast, another study conducted by Gardner^13^ on a different group of rhesus monkeys did not demonstrate any activity at that suture during RME. FEM studies^15,16,17^ have suggested that the zygomaticomaxillary sutures are places of compressive forces during palatal expansion. The results of this study show an average of 1.2 mm of sutural displacement at both the superior and inferior borders of these sutures, which is more consistent with the results that Starnbach^10^ showed histologically. Previous RME studies carried out with children did not assess zygomaticomaxillary sutures, most likely due to the difficulty in identifying the suture on posteroanterior or lateral cephalograms. When the individual planes are evaluated, the superior borders of the zygomaticomaxillary suture move transversely by an average of 1.54 mm, but the borders move slightly downward (0.1 mm) and forward (0.88 mm) as well. The inferior borders of the of the zygomaticomaxillary suture also move transversely by an average of 1.32 mm, and forward by an average of 1.82 mm. These findings are consistent with the accepted fact that the entire maxilla moves down and forward with RME.

The frontonasal suture proved to have a significant amount of displacement, with an average displacement of only 0.6 mm. The movement of the borders of this suture was also in a down and forward direction, as expected. However, other studies have suggested that the greatest response to maxillary expansion outside of the oral cavity is found at this suture.^12^

The intermaxillary suture is usually included as an extension of the midpalatal suture, since it appears to be continuous with that suture. The displacement of the intermaxillary suture was measured at its most apical landmark, the ANS. It is interesting to note that this is the only suture with significant displacement which had a significant correlation with the amount of expansion of the appliance. The only significant movement was in a transverse direction at ANS.

Assessment of A point showed a significant displacement backward. A point also had an average displacement of nearly 3 mm downward, but this was not statistically significant. Additionally, PNS had an average downward displacement of about 1 mm, which was not statistically significant. Studies have shown contradictory results regarding the tipping of the palatal plane, with some studies showing that the posterior tipping was greater than that at the anterior aspect, while other studies show no significant tipping. Even with some relatively large downward measurements, this study proved that the tipping of the palatal plane is not significant with RME.

The lack of significant change in the transpalatal suture appears to confirm a prior study^7^ carried out with dried skulls and human patients in which the palatine bones do not separate from the maxillary bones under the forces of palatal expansion. Timms^7^ proposed it was the connection to the sphenoid bone that prevented the sutural displacement and the pterygoid processes from simply bending in response to the expansion forces. While the separation of the transpalatal suture was not statistically significant, there was some separation in some patients. A larger sample size may be able to refute or confirm these results.

Only the external surfaces of sutures were examined during this study. The changes that occurred during RME were recorded in all three planes of space. The element of time was disregarded, since there was, on average, only about 3 weeks between T_0_ and T_1_. While the subjects were all growing children, the amount of growth that occurs over 3 weeks is negligible.

The RME appliance for this study was directly attached to the maxillary first molars, and one would expect that the molars would tip buccally as a result of the expansion forces. While there were individual variations, the molars did as predicted with about a 10 degree increase in the intermolar angle as a result of RME.

This study has established the fact that circummaxillary sutures are affected by RME in growing children, and that the movements of facial bones can be reliably quantified in three planes of space using CBCT. The overall forward and downward movement of the maxilla can be seen, as well as the transverse changes. Individual assessment of the changes occurring at the maxillary molars can also be performed. The results of this study provide a link among the prior studies carried out with non-human primates, dry skulls and computer models, and describe what actually occurs in the sutures in patients, as the result of RME treatment.

## CONCLUSIONS

Rapid maxillary expansion results in significant displacement of the frontonasal, intermaxillary, zygomaticomaxillary and midpalatal sutures in growing children in all three planes of space.

## References

[1] Isaacson RJ, Ingram AH (1964). Forces produced by rapid maxillary expansion. Angle Orthod.

[2] Proffit WR (2000). Contemporary Orthodontics.

[3] Bell RA (1982). A review of maxillary expansion in relation to rate of expansion and
patients age. Am J Orthod.

[4] Haas AJ (1961). Rapid expansion of the maxillary dental arch and nasal cavity by
opening the midpalatal suture. Angle Orthod.

[5] Bishara SE, Staley RN (1987). Maxillary expansion: clinical implications. Am J Orthod Dentofacial Orthop.

[6] Chaconas SJ, Caputo AA (1982). Observation of orthopedic force distribution produced by maxillary
orthodontic appliances. Am J Orthod.

[7] Timms DJ (1980). A study of basal movement with rapid maxillary
expansion. Am J Orthod.

[8] Baydas B, Yavuz I, Uslu H, Dagsuyu IM, Ceylan I (2006). Nonsurgical rapid maxillary expansion effects on craniofacial
structures in young adult females. A bone scintigraphy study. Angle Orthod.

[9] Habersack K, Karoglan A, Sommer B, Benner KU (2007). High-resolution multislice computerized tomography with multiplanar
and 3-dimensional reformation imaging in rapid palatal expansion. Am J Orthod Dentofacial Orthop.

[10] Starnbach HK, Bayne DI, Cleall JF, Subtelny JD (1966). Facioskeletal and dental changes resulting from rapid maxillary
expansion. Angle Orthod.

[11] Cleall JF, Bayne DI, Posen JM, Subtelny JD (1965). Expansion of the midpalatal suture in the monkey. Angle Orthod.

[12] Starnbach HK, Cleall JF (1964). The Effects of splitting the midpalatal suture on the surrounding
structures. Am J Orthod.

[13] Gardner GE, Kronman JH (1971). Cranioskeletal displacements caused by rapid palatal expansion in the
rhesus monkey. Am J Orthod.

[14] Marcotte MR (1977). The instantaneous transverse changes in the maxilla due to different
points of force application. J Dent Res.

[15] Jafari A, Shetty KS, Kumar MK (2003). Study of stress distribution and displacement of various craniofacial
structures following application of transverse orthopedic forces: a
three-dimensional FEM study. Angle Orthod.

[16] Boryor A, Geiger M, Hohmann A, Wunderlich A, Sander C, Martin Sander F (2008). Stress distribution and displacement analysis during an intermaxillary
disjunction: a three-dimensional FEM study of a human skull. J Biomech.

[17] Gautam P, Valiathan A, Adhikari R (2007). Stress and displacement patterns in the craniofacial skeleton with
rapid maxillary expansion: a finite element method study. Am J Orthod Dentofacial Orthop.

[18] Akkaya S, Lorenzon S, Ucem TT (1999). A comparison of sagittal and vertical effects between bonded rapid and
slow maxillary expansion procedures. Eur J Orthod.

[19] Cameron CG, Franchi L, Baccetti T, McNamara JA (2002). Long-term effects of rapid maxillary expansion: a posteroanterior
cephalometric evaluation. Am J Orthod Dentofacial Orthop.

[20] Chang JY, McNamara JA, Herberger TA (1997). A longitudinal study of skeletal side effects induced by rapid
maxillary expansion. Am J Orthod Dentofacial Orthop.

[21] Chung C-H, Font B (2004). Skeletal and dental changes in the sagittal, vertical, and transverse
dimensions after rapid palatal expansion. Am J Orthod Dentofacial Orthop.

[22] Cross DL, McDonald JP (2000). Effect of rapid maxillary expansion on skeletal, dental, and nasal
structures: a postero-anterior cephalometric study. Eur J Orthod.

[23] Silva OG, Boas MC, Capelozza L (1991). Rapid maxillary expansion in the primary and mixed dentitions: a
cephalometric evaluation. Am J Orthod Dentofacial Orthop.

[24] Davis WM, Kronman JH (1969). Anatomical changes induced by splitting of the midpalatal
suture. Angle Orthod.

[25] Garrett BJ, Caruso JM, Rungcharassaeng K, Farrage JR, Kim JS, Taylor GD (2008). Skeletal effects to the maxilla after rapid maxillary expansion
assessed with cone-beam computed tomography. Am J Orthod Dentofacial Orthop.

[26] Krebs A (1958). Expansion of the midpalatal suture studied by means of metallic
implants. Eur Orthod Soc Rep.

[27] Krebs A (1964). Midpalatal suture expansion studies by the implant method over a
seven-year period. Eur Orthod Soc Rep.

[28] Lione R, Ballanti F, Franchi L, Baccetti T, Cozza P (2008). Treatment and posttreatment skeletal effects of rapid maxillary
expansion studied with low-dose computed tomography in growing
subjects. Am J Orthod Dentofacial Orthop.

[29] Wertz RA (1970). Skeletal and dental changes accompanying rapid midpalatal suture
opening. Am J Orthod.

[30] Phatouros A, Goonewardene MS (2008). Morphologic changes of the palate after rapid maxillary expansion: a
3-dimensional computed tomography evaluation. Am J Orthod Dentofacial Orthop.

[31] Felippe NLO, Silveira AC, Viana G, Kusnoto B, Smith B, Evans CA (2008). Relationship between rapid maxillary expansion and nasal cavity size
and airway resistance: short- and long-term effects: short- and long-term
effects. Am J Orthod Dentofacial Orthop.

[32] Rungcharassaeng K, Caruso JM, Kan JY, Kim J, Taylor G (2007). Factors affecting buccal bone changes of maxillary posterior teeth
after rapid maxillary expansion. Am J Orthod Dentofacial Orthop.

[33] Podesser B, Williams S, Crismani AG, Bantleon HP (2007). Evaluation of the effects of rapid maxillary expansion in growing
children using computer tomography scanning: a pilot study. Eur J Orthod.

[34] Palomo JM, Kau CH, Palomo LB, Hans MG (2006). Three-dimensional cone beam computerized tomography in
dentistry. Dent Today.

[35] Kau CH, Richmond S, Palomo JM, Hans MG (2005). Three-dimensional cone beam computerized tomography in
orthodontics. J Orthod.

[36] Lagravère MO, Hansen L, Harzer W, Major PW (2006). Plane orientation for standardization in 3-dimensional cephalometric
analysis with computerized tomography imaging. Am J Orthod Dentofacial Orthop.

[37] Pett MA (1997). Nonparametric statistics for health care research; statistics for small
samples and unusual distributions.Thousand Oaks,.

